# Species Tree Inference with BPP Using Genomic Sequences and the Multispecies Coalescent

**DOI:** 10.1093/molbev/msy147

**Published:** 2018-07-23

**Authors:** Tomáš Flouri, Xiyun Jiao, Bruce Rannala, Ziheng Yang

**Affiliations:** 1 Department of Genetics, Evolution and Environment, University College London, London, United Kingdom; 2 Department of Ecology and Evolution, University of California, Davis, CA

**Keywords:** BPP, MCMC, multispecies coalescent, species tree inference

## Abstract

The multispecies coalescent provides a natural framework for accommodating ancestral genetic polymorphism and coalescent processes that can cause different genomic regions to have different genealogical histories. The Bayesian program BPP includes a full-likelihood implementation of the multispecies coalescent, using transmodel Markov chain Monte Carlo to calculate the posterior probabilities of different species trees. BPP is suitable for analyzing multilocus sequence data sets and it accommodates the heterogeneity of gene trees (both the topology and branch lengths) among loci and gene tree uncertainties due to limited phylogenetic information at each locus. Here, we provide a practical guide to the use of BPP in species tree estimation. BPP is a command-line program that runs on linux, macosx, and windows. This protocol shows how to use both BPP 3.4 (http://abacus.gene.ucl.ac.uk/software/) and BPP 4.0 (https://github.com/bpp/).

## Introduction

In the past decade, it has become evident that different genes or genomic regions may have different evolutionary histories (gene trees), due to several important biological processes, including the coalescent process in ancestral species, gene duplication, and horizontal gene transfer (introgression) (Maddison 1997; Nichols 2001; Edwards 2009). Gene tree heterogeneity due to the coalescent process is universal as it is the natural consequence of polymorphism and genetic drift in ancestral species, or the stochastic nature of the coalescent process. However, the magnitude of the differences depends on population genetic parameters. Conflicts between gene trees and the species tree caused by the coalescent processes in ancestral species are often referred to as incomplete lineage sorting (ILS). ILS is most prominent if the species arose in a rapid succession of speciation events (a species radiation), creating short internal branches on the species tree, and if the ancestral species had large population sizes. For such challenging phylogenetic problems, traditional phylogenetic methods, which concatenate the sequences across loci and infer a common phylogeny using maximum likelihood or Bayesian inference, may be statistically inconsistent and converge to an incorrect phylogeny when the number of loci increases (Kubatko and Degnan 2007; Edwards et al. 2016). Similarly conducting a separate phylogenetic analysis at each locus and using the most common gene tree as the species tree estimate can also be inconsistent (Degnan and Rosenberg 2006, 2009).

**Fig. 1.**
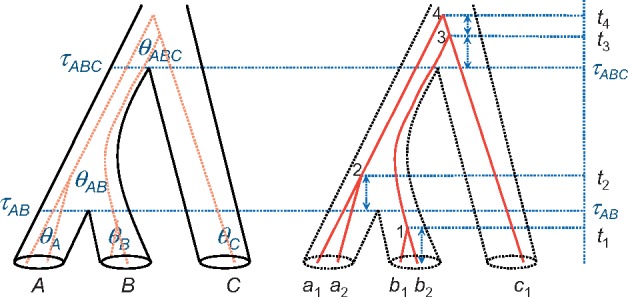
A species tree for three species (*A*, *B*, and *C*) to illustrate the parameters of the MSC model, with a gene tree for five sequences (*a*_1_ and *a*_2_ sampled from species *A*, *b*_1_ and *b*_2_ from species *B*, and *c*_1_ from species *C*) running inside the species tree. Within each species/population, sequences coalesce at random at the rate determined by the population size (or *θ* parameter), generating a gene tree with branch lengths (coalescent times), conditioned on the species tree. Note that *θ_C_* is not estimable if there is at most one sequence from species *C* at each locus.

The multispecies coalescent (MSC) has emerged as the natural framework to account for genealogical heterogeneity across the autosomal genome due to ILS (Rannala and Yang 2003; Edwards 2009; Xu and Yang 2016). The MSC lies at the interface of population genetics and molecular phylogenetics (Rannala and Yang 2003; see also Takahata et al. 1995; Yang 2002). It differs from models of population structure and subdivision in population genetics in that it accounts for the history of species/population divergences. It differs from traditional phylogenetic models in that it accounts for the coalescent process and the resulting genealogical heterogeneity across the genome. Because it accounts for the coalescent process in both extant and extinct ancestral species, the MSC naturally accommodates ILS (fig. 1).

The basic MSC model for a species tree of *s* species involves two types of parameters: *s*−1 species divergence times (*τ*s) and up to 2*s*−1 population size parameters for the populations on the species tree (*θ*s) (fig. 1). Both *τ* and *θ* parameters are measured by the sequence distance or the expected number of mutations/substitutions per site. The parameter *θ* = 4*Nμ* is the average distance between two sequences sampled at random from a population with effective population size *N*, where *μ* is the mutation rate per site per generation. For the example, for the human species, *θ* ≈ 0.0006, meaning that two genomic sequences from the species have on average 0.6 differences per kb. The parameter *τ* is the age of an internal node (species divergence event) in the species tree, measured in units of expected number of mutations per site. For example, in the species tree, ((A, B), C), there are two species divergence times (*τ*_AB_ and *τ*_ABC_) and five population sizes (*θ*_A_, *θ*_B_, *θ*_C_, *θ*_AB_, and *θ*_ABC_). Note that at least two sequences are needed to calculate a distance (or to estimate *θ*); if only one sequence is available for an extant (contemporary) species at every locus, *θ* for that species cannot be estimated.

Two kinds of methods are often used to estimate the species tree under the MSC: the summary and full-likelihood methods (Edwards et al. 2016; Xu and Yang 2016). The summary methods typically have two steps: 1) estimating the gene tree at each locus using phylogenetic methods and 2) treating the estimated gene trees as observed data to infer the species tree. They tend to have reduced statistical efficiency but are computationally fast. The full likelihood methods operate on sequence alignments and have the strength of accommodating uncertainties in gene trees. By combining information across many loci, those methods can produce a confident and reliable species tree estimate even if there are few variable or informative sites at each locus so that the information at every locus is weak (Xu and Yang 2016; Shi and Yang 2018).

In this protocol, we assume some familiarity with Bayesian MCMC and molecular phylogenetics, or experience with phylogenetic programs such as MrBayes. For an introduction to Bayesian MCMC algorithms, see Nascimento et al. (2017). For detailed reviews of the MSC model and Bayesian inference under the MSC, see Yang (2014: Chapter 9), Rannala (2015), Edwards et al. (2016), Mallo and Posada (2016), and Xu and Yang (2016).

## Species Tree Inference Using BPP

The BPP implementation of the MSC model uses the Bayesian model-selection framework to evaluate different models of species delimitations and species phylogenies. The basic assumptions include the following: 1) no recombination within a locus, 2) free recombination between loci, 3) no migration (gene flow) between species, 4) neutral evolution, and 5) clock-like evolution. These assumptions suggest that certain properties are desirable for data sets to be analyzed using BPP. To satisfy assumption i short genomic segments (e.g., 500 to 1,000 bp) should be used (called loci); this insures that recombination within a locus is rare. To satisfy assumption ii the different loci should be physically distant from one another in the genome; this insures that recombination between them is common, allowing the loci to have approximately independent histories. To satisfy assumption iii the populations for analysis should not be experiencing significant ongoing gene flow; this assumption can be tested using preliminary population genetic analyses (Reich et al. 2010; Dalquen et al. 2017). To satisfy assumption iv, the loci should be evolving neutrally, implying that their gene trees are not affected to a significant extent by natural selection. Despite this assumption, protein-coding genes appear to be useable for BPP analysis even if they show obvious evidence of purifying selection. Most proteins perform similar functions in closely related species and the main effect of purifying selection on nonsynonymous mutations is a reduction of the neutral mutation rate for the locus. Studies comparing species trees inferred using exons and using introns or noncoding DNA gave highly consistent results between the two kinds of data (Shi and Yang 2018). Nevertheless, it is prudent to analyze noncoding and coding regions of the genome as separate data sets. Assumption v arises because BPP currently uses the JC69 model (Jukes and Cantor 1969) to correct for multiple mutations/substitutions at a site with a constant rate over time (the molecular clock). The program is thus suitable for analyzing closely related species with sequence divergences below ∼10%.

BPP is a Bayesian Markov chain Monte Carlo (MCMC) program for analyzing multilocus sequence data under the MSC. It can be used for four kinds of inference problems or analyses (Yang 2015):

- A00 (specified by speciesdelimitation=0 and speciestree=0): estimation of parameters under the MSC (including species divergence times and population sizes) on a fixed species phylogeny (Yang 2002; Rannala and Yang 2003; Burgess and Yang 2008);
- A01 (speciesdelimitation=0 and speciestree=1): estimation of species phylogeny when species assignment and delimitation is given (Yang and Rannala 2014; Rannala and Yang 2017);
- A10 (speciesdelimitation=1 and speciestree=0): comparison of species delimitation models induced on a given “guide” tree (Yang and Rannala 2010; Rannala and Yang 2013);
- A11 (speciesdelimitation=1 and speciestree=1): joint comparison of species delimitation/assignement and species tree models (Yang and Rannala 2014; Rannala and Yang 2017).

In this protocol, we focus on analysis A01: species tree estimation.

## Protocol

Here, we describe the procedure for installing or compiling and running BPP from the command line in either Linux, Windows, or Mac OSX. If you have not used the command line before, please work through one of the following short tutorials first:

http://abacus.gene.ucl.ac.uk/software/CommandLine.Windows.pdf

http://abacus.gene.ucl.ac.uk/software/CommandLine.MACosx.pdf

### Obtaining and Compiling the BPP Program

The protocols presented here use both BPP versions 3.4 and 4.0. Both programs are written in the C language and can be compiled to run on Linux, MacOSX, and windows. BPP 3.4 is available at http://abacus.gene.ucl.ac.uk/software/, and the manual (BPPDOC.pdf) is included in the release, which details the format of the data files, the screen output, as well as the interpretation of the output. BPP 4 is a highly optimized reimplementation, available at https://github.com/bpp/. On-line documentation is available for BPP 4 at https://github.com/bpp/wiki. Precompiled Windows and Mac OS X executables are available for BPP 3.4 and 4 from their respective websites. For linux you may need to compile the programs yourself to generate the executable file bpp (optionally you can manually compile the program executable for Mac OS X as well if you have the X Code compiler system installed).

The program compilation needs to be done only once. Here, it is assumed that you have uncompressed the distribution file into a subdirectory bpp. On Linux, for example, the following commands use the gcc compiler to compile the program and move the generated executable file (bpp) into the bin/folder.

**Table:** 

BPP3.4	BPP4.0
cd bpp	cd bpp
mkdir bin	mkdir bin
cd src	cd src
gcc -o bpp -O3 bpp.c tools.c –lm	make
mv bpp ../bin	mv bpp ../bin

### Running BPP

**Fig. 2.**
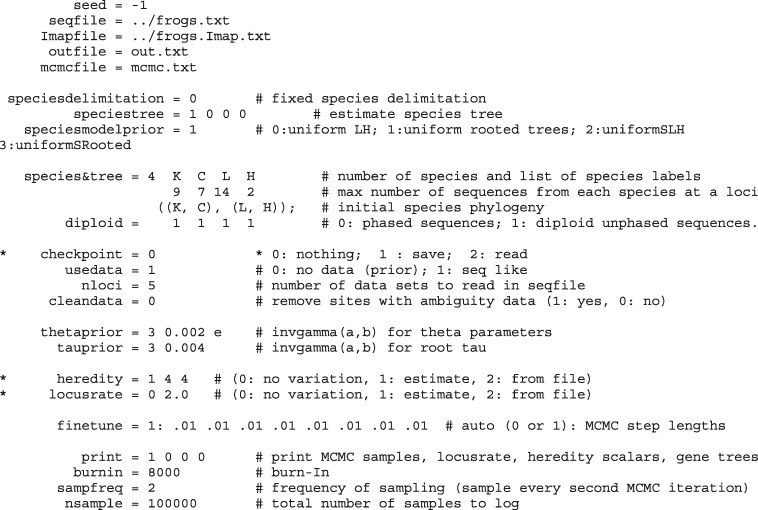
Sample control file A01.bpp.ctl for species tree estimation (with speciesdelimitation=0 and speciestree=1). Lines starting with an asterisk are comments and the default values of speciesdelimitation and speciestree are 0.

**Fig. 3.**
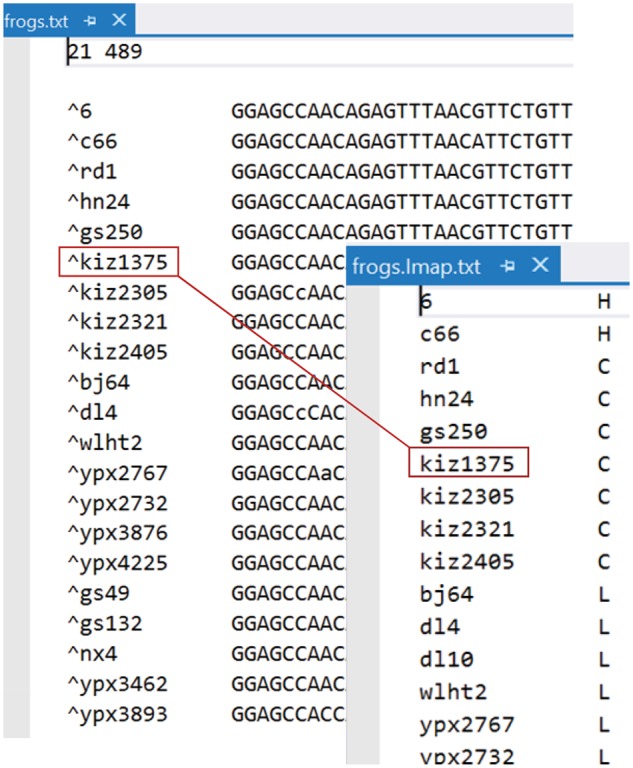
The sequence data file (frogs.txt) and the Imap file (frogs.Imap.txt). In the sequence file each sequence is tagged with an individual/specimen ID (such as kiz1375, for Kunming Institute of Zoology #1375). The part of the sequence name before the caret (^) is read and then ignored. In the Imap file each individual is assigned to a species/population: for example, individual kiz1375 is assigned to species C.

BPP takes three input data files: 1) a control file that specifies the model and the priors and effectively “drives” the analysis (fig. 2), 2) a sequence alignment file that contains the sequence data for all loci, and 3) an individual-to-population map file (Imap file) that assigns each individual to a population (fig. 3). All those are plain text files and can be prepared using any text editor. Here, we will use a data set of five nuclear loci from the East Asia brown frogs (Zhou et al. 2012), previously analyzed by Yang (2015). The three input files are in the folder frogs in the release. We will run each analysis twice in two folders, frogs/r1/and frogs/r2/. Start two command-line terminals. Then change directory to r1 (or r2), and run the program as follows.

**Table:** 

On Windows	On Linux/Unix/Mac OSX
To run BPP3.4	
cd frogs\r1..\..\bin\ bpp..\A01. bpp .ctl	cd frogs/r1 ../../bin/ bpp ../A01. bpp .ctl
To run BPP4.0	
cd frogs\r1..\..\bin\bpp –cfile=..\A01.bpp.ctl	cd frogs/r1 ../../bin/ bpp –cfile=../A01. bpp .ctl

Here, A01.bpp.ctl is the control file for analysis A01 (fig. 2). The input data file names are specified in the control file. Note that in the control file, the data file is specified as ./frogs.txt instead of frogs.txt, because the file is in the frogs folder while we run BPP in the frogs/r1/folder.

The run will produce an MCMC sample file (mcmc.txt), which is read and summarized by BPP to produce the output file (out.txt). Consistency across runs is an important indicator of MCMC convergence so we recommend running the same analysis multiple times. If the multiple runs produce similar posterior, MCMC samples from the multiple runs may be merged and then summarized: Append one MCMC sample file to the end of another (and remove the header line of the second file in the case of A00 analysis). Then run BPP with print=−1.

### The Imap File

The Imap file assigns individuals or sequences to the populations or species. In the sequence data file, each sequence name has a tag (following the caret ^) which is interpreted as an individual ID and used in the Imap file to assign the sequence to a population (fig. 3). After this information is read, BPP uses the population IDs and ignores the individual IDs. In theory it would be sufficient to tag each sequence by its population ID without the need for the Imap file. The current two-layer design makes it easy to change the assignments, which involves editing the small Imap file without altering the much larger sequence file.

### The Sequence Alignment File

Sequence alignments for multiple loci are in the phylip/paml format (fig. 3), with one alignment following the other, all in one file. The number of loci (or alignments) is specified by the variable nloci in the control file: BPP reads the specified number of loci and ignores the rest of the file.

### The Control File

The variables in the control file (fig. 2) are described in detail in the program documentation. Here, we focus on those important to this protocol.

Analysis A01 (species tree estimation) is triggered by using speciesdelimitation=0, speciestree=1. BPP uses the subtree pruning and regrafting (SPR) and NodeSlider algorithms to change the species tree topology in the MCMC (Yang and Rannala 2014; Rannala and Yang 2017), with the species tree in the control file used as the starting species tree. The posterior distribution should be independent of the starting species tree. If the posterior results look too different between runs that started from different starting species trees, we need to rerun the program using a larger number of samples (nsample) and/or larger sampling frequency (sampfreq).

The diploid variable indicates whether the sequences from each species are phased (0) or unphased (1). If this line is missing, all sequences in the sequence data file are assumed to be phased. If the indicator is 1 for a species, all sequences from that species are assumed to be diploid unphased data. BPP does not allow some sequences from a species to be phased while others from the same species to be unphased. In an unphased sequence, a heterozygote site is represented by using the ambiguity characters Y, R, M, K, S, and W. For example, a Y in an unphased sequence means a T/C heterozygote, but in a phased sequence, it means an ambiguity state that is either T or C. BPP handles unphased sequences by analytically integrating over different phase resolutions of the heterozygote sites in the likelihood calculation, using the approach of Gronau et al. (2011). This works for a small number of sequences per locus and may not be computationally feasible when there are many sequences at each locus.

In species tree estimation (A01 analysis), one has to specify a prior distribution for the different species tree topologies and also priors for parameters (*θ*s and *τ*s) in each species tree. Two priors for species trees are available in BPP. Prior 0 (speciesmodelprior = 0) assigns equal probabilities for the labeled histories (which are rooted trees with the internal nodes ordered by relative age), while Prior 1 (speciesmodelprior = 1) assigns equal probabilities for the rooted trees (Yang and Rannala 2014). For instance, there are 15 rooted trees in the case of four species (A, B, C, and D), with 12 unbalanced and 3 balanced trees. Each unbalanced tree, for example, (((A, B), C), D), is compatible with only one labeled history as there is only one ordering of the internal nodes. Each balanced tree, for example, ((A, B), (C, D)), is compatible with two labeled histories, depending on whether the ancestor of A and B is older or younger than the ancestor of C and D. Prior 0 assigns the probability 1/18 to each of the unbalanced trees and 2/18 to each of the balanced trees. Prior 1 assigns the probability 1/15 to each of the 15 rooted trees. We use Prior 1, which is the default.

Within each species tree model, we assign the inverse-gamma priors *θ* ∼ IG(3, 0.002) for all *θ*s and *τ* ∼ IG(3, 0.004) for the age of the root (*τ*_0_). The inverse-gamma IG(*α*, *β*) has mean *m* = *β*/(*α*−1) if *α* > 1 and variance *s*^2^ = *β*^2^/[(*α*−1)^2^(*α*−2)] if *α* > 2. If little information is available about the parameters, you can use *α* = 3 for a diffuse prior and then adjust *β* so that the mean looks reasonable. For example, parameter *θ* measures the genetic diversity (heterozygosity) in the species. This varies among species, with 0.01 (one difference per 100 bp) to be a large value while 0.001 a small one. Parameter *τ*_0_ measures the age of the root in the rooted species tree and depends on the species included in the data set. Thus including an outgroup species will typically mean that a larger prior mean for *τ*_0_ is appropriate.

It is useful to plot the inverse-gamma density and calculate the 95% prior interval. The R functions for doing this are in the MCMCpack, so install the package first:

install.packages(“invgamma”);

Then

library(“invgamma”);

a=3;b=0.002;

curve(dinvgamma(x, a, b),from=0,to=0.01)

qinvgamma(c(0.025, 0.975), a, b)

**Fig. 4.**
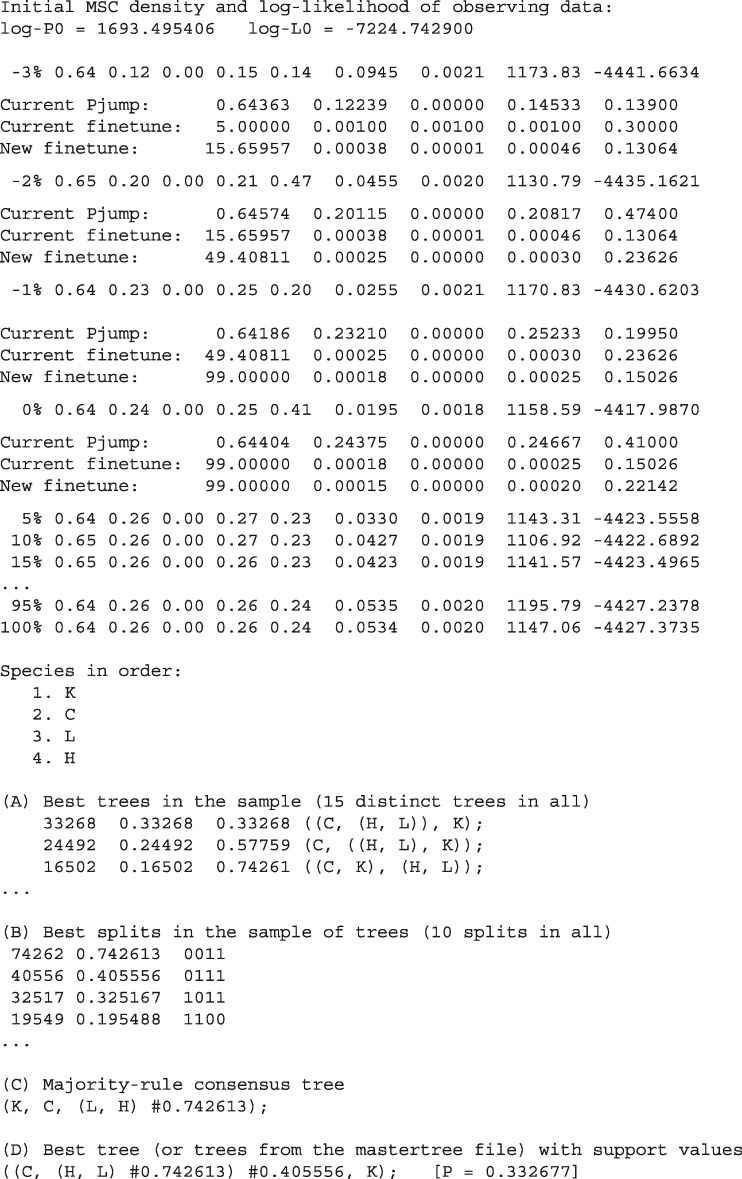
Output from BPP for analysis A01 (species tree estimation). The progress indicator is negative during the burnin, and BPP goes through four rounds of automatic step-length adjustments, aiming to achieve a near-optimal acceptance proportion of 30% for the parameter-moves (Yang and Rodríguez 2013). Sampling (in mcmc.txt) starts after the burn-in is over. At the end of the MCMC run, the sample is processed to calculate the posterior probabilities of the species trees (section A), which are further summarized to calculate the posterior for splits as well as the majority-rule consensus tree.

BPP collects the species trees (as well as parameters *θ*s and *τ*s) into the sample file mcmc.txt. At the end of the MCMC run, it summarizes the MCMC sample to produce the output file out.txt (fig. 4). This is self-explanatory.

Both BPP 3.4 and 4.0 implement an option of integrating out analytically the *θ* parameters, using conjugate inverse-gamma priors (Hey and Nielsen 2007). This reduces the state of the Markov chain, resulting in slight improvement in the mixing properties of cross-model MCMC algorithms. It is advisable to use this option in species tree estimation (the A01 analysis). The option can be turned off so that the posteriors for *θ*s are generated in the A00 analysis to estimate parameters on a fixed species tree, such as the maximum posterior probability (MAP) species tree.

### The Example Data Set from East Asian Brown Frogs

We use as our example the five nuclear loci from East Asian brown frogs in the *Rana chensinensis* species complex (Zhou et al. 2012). There are 21–30 sequences per locus, and the sequences are 285–489 bp long. Three morphologically recognized species exist in this group: *R. chensinensis* (clades C and L), *R. kukunoris* (K), and *R. huanrensis* (H), but *R. chensinensis* has a widespread distribution, with two populations (C and L) recognized. The sequences are unphased but were treated (incorrectly) as phased in the tutorial of Yang (2015). Here, we will use the diploid option in BPP 3.4 and 4.0 to reanalyze those data. The three input files are in the folder frogs. We will run each analysis twice in two folders, frogs/r1/and frogs/r2/. Start two command-line terminals. Then change directory to r1 (or r2), and run the program as follows.

**Fig. 5.**
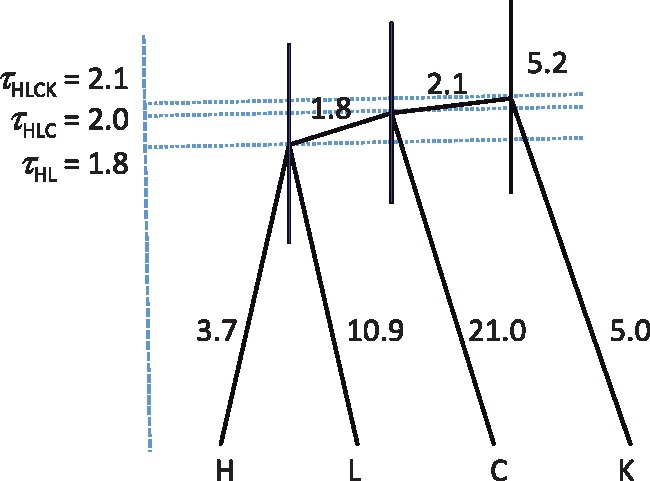
The MAP species tree for four brown frog species/populations, showing the parameter estimates (×10^−3^). The branch lengths represent the posterior means of divergence times (*τ*s), while the node bars show the 95% HPD intervals. Posterior means of *θ*s are shown along the branches. The priors used in the analysis are *θ* ∼ IG(3, 0.002) for all populations and *τ*_KCLH_ ∼ IG(3, 0.004) for the root age. The tree is drawn with FigTree using the BPP output FigTree.tre.

First, we run the A01 analysis to estimate the species tree, with the *θ* parameters integrated out analytically (using the control file A01.bpp.ctl with thetaprior=30.002 without the “e”). The MAP species tree, which is the (binary) species tree that has the maximum posterior probability (or has been visited most frequently by the MCMC algorithm), is shown in figure 5: (((LH)C)K). The MAP tree has only 32% posterior probability, indicating that the five loci have only weak phylogenetic information. The tree differs from the species tree inferred by Yang (2015, fig. 2): ((KC)(LH)), although the support is weak in both analyses. The majority-rule consensus species tree, which shows only splits found in at least half of the sampled species trees, is a star tree (fig. 4). Note that if the consensus species tree is binary, it must be the MAP tree, but otherwise the two are different.

Second, we run the A00 analysis with the species tree fixed at the MAP tree to estimate the parameters (*τ*s and *θ*s) of the MSC model (using the control file A00.bpp.ctl with thetaprior=30.002e, with the “e”). The posterior means are shown in figure 5. It is noteworthy that the diploid option affects parameter estimates. In particular, the estimates of *θ* are greater now, because in the analysis of Yang (2015), heterozygous nucleotides in the unphased sequences were incorrectly treated as ambiguity characters.

We changed the inverse gamma priors for *θ*s and *τ*_0_ to evaluate their impact on the posterior probabilities for species trees. We used the shape parameter *α* = 3 in the inverse-gamma as a diffuse prior, and *α* = 21 as an informative prior, and then varied the prior mean by two orders of magnitude. Note that the mean of the inverse gamma IG(*α*, *β*) is *β*/(*α*−1) and the coefficient of variation is $\sqrt{1/\left(\alpha -2\right)}$, with *α* > 2. The results are summarized in table 1. As the data set with five loci is small, the prior had substantial impact on the posterior. Having very large *θ*s in the prior tends to reduce posterior probabilities for the MAP tree, apparently because large *θ*s allow the poor species trees to “explain” the data easily by attributing the conflicting gene trees to random fluctuations of the coalescent process in the ancestors. Having a very small *τ*_0_ in the prior tends to reduce posterior probabilities for the MAP tree as well, possibly because a small *τ*_0_ causes the different species trees to look similar, pushing all coalescent events into the root species.

**Table 1.** Posterior Probability for Clades in the MAP Tree under Different Priors for the Frogs Dataset

Prior		(HL)	(HLC)	Tree	*P*_jump_
*θ* ∼ IG(3, 0.0002)	*τ*_0_ ∼ IG(3, 0.004)	0.69	0.35	0.28	6.6%
***θ* ∼ IG(3, 0.002)**	***τ*_0_ ∼ IG(3, 0.004)**	**0.72**	**0.38**	**0.32**	**5.6%**
*θ* ∼ IG(3, 0.02)	*τ*_0_ ∼ IG(3, 0.004)	0.45	0.30	0.19	10.0%
*θ* ∼ IG(21, 0.002)	*τ*_0_ ∼ IG(3, 0.004)	0.41	0.34	0.20	12.3%
*θ* ∼ IG(21, 0.02)	*τ*_0_ ∼ IG(3, 0.004)	0.47	0.36	0.24	10.0%
*θ* ∼ IG(21, 0.2)	*τ*_0_ ∼ IG(3, 0.004)	0.39	0.35	0.19	14.1%
*θ* ∼ IG(3, 0.002)	*τ*_0_ ∼ IG(3, 0.0004)	0.69	0.33	0.28	6.3%
***θ* ∼ IG(3, 0.002)**	***τ*_0_ ∼ IG(3, 0.004)**	**0.72**	**0.38**	**0.32**	**5.6%**
*θ* ∼ IG(3, 0.002)	*τ*_0_ ∼ IG(3, 0.04)	0.69	0.53	0.44	4.6%
*θ* ∼ IG(3, 0.002)	*τ*_0_ ∼ IG(21, 0.004)	0.70	0.41	0.30	7.5%
*θ* ∼ IG(3, 0.002)	*τ*_0_ ∼ IG(21, 0.04)	0.69	0.34	0.29	6.2%
*θ* ∼ IG(3, 0.002)	*τ*_0_ ∼ IG(21, 0.4)	0.87	1.00	0.87	0.7%

Note.—The MAP tree is (((HL)C)K), shown in figure 5. *P*_jump_ is the acceptance proportion of the SPR move across species trees recorded during the MCMC run. The same analysis was run three times (with burnin = 8000, sampfreq = 2 and nsample = 10^5^) to check for consistency between runs, and the combined sample was then summarized. There were mixing problems for the last analysis, with priors θ ∼ IG(3, 0.002) and τ_0_ ∼ IG(21, 0.4), in which case we used burnin = 4 × 10^4^ and nsample = 5 × 10^5^.

## Discussion

### The Choice of Sequence Loci

The ideal data for analyses using BPP are loosely linked short genomic segments (referred to as “loci”) (Takahata et al. 1995; Burgess and Yang 2008; Shi and Yang 2018). A major biological factor that the MSC model accommodates is the genealogical heterogeneity across genome: that is, different regions of the autosomal genome have different gene tree topologies and branch lengths (coalescent times). While the mutational rate may also vary along the genome, this variation is expected to have much less impact on the species tree inference, especially when the species are close and the sequences are similar. Thus multiple genes from the mitochondrial (or chloroplast) genome should be treated as one “locus” in the MSC-based analysis. Typically the mitochondrial genome has a different mutation rate and different effective population size from the autosomal genome. BPP has a control variable (locusrate) for rate variation among loci and another variable (heredity) for different heredity scalars for population sizes (Burgess and Yang 2008), so that in theory the program allows joint analysis of autosomal and mitochondrial loci in one data set. However, certain biological processes such as introgression and selection may affect the autosomal and mitochondrial genomes differently, and it may be useful to analyze the autosomal and mitochondrial loci as separate data sets to examine possible heterogeneity in the species tree.

The MSC model assumes that the sequences from the different species are random samples. Thus all sequences from a particular species (or a random sample of them) should be used: for example, it is not advisable to use only the distinct haplotypes because removal of the identical sequences leads to underestimation of *θ*s. Similarly one should not filter loci based on bootstrap support values; using only loci with high phylogenetic information content will bias estimates.

### The Limits of BPP

The question is often asked what are were the limits on the size of data sets that can be analyzed by BPP? The short answer is that many modern data sets are too large to be simultaneously analyzed using BPP in a reasonable time period (hours or days). The computational demands increase with an increase in either the number of species, the number of loci, the number of sequences per locus, or the number of sites per sequence. Increasing the number of species increases both the number of possible species trees and the number of parameters on each species tree so that the parameter space becomes much larger. As in conventional phylogenetic inference the number of rooted species trees increases explosively with the number of species (*s*). The number of parameters on each species tree (*s*−1 + 2 *s*−1 = 3 *s*−2) increases linearly with *s*. Increasing the number of loci should also have a near linear effect on the computation for each MCMC iteration. However a compounding factor is that with more loci, the posterior for the parameters (*θ*s and *τ*s) in each species tree become highly concentrated, making it more difficult for the algorithm to move from one species tree to another; this can create mixing problems. Increasing the number of sequences adds to the size of the gene trees, as well as the number of variables (such as coalescent times) to update during the MCMC. The number of sites per sequence has the least impact in terms of computational expense.

Finally the type of analysis also matters. The A00 analysis generates the posterior distribution of the parameters (*θ*s and *τ*s) when the species tree and the MSC model is fixed. The MCMC algorithm implemented in the BPP program for this inference has been successfully applied to genomic data sets of >50,000 loci (Burgess and Yang 2008). The other three analyses (A01, A10, and A11), including analysis A01 discussed in this protocol, are transmodel inferences (in the terminology of Green 2003), in which the Markov chain moves between different models, each of which is an instance of the MSC model, although the number of species and the species phylogeny may differ among the models. The main objective of the transmodel inference is the calculation of posterior model probabilities. Poor mixing is a common problem with transmodel algorithms.

For species tree estimation (the A01 analysis), BPP has been used to analyze data sets of 919 loci (19 sequences per locus, median length 706 bp) from Philippine shrews (genus *Crocidura*) (Giarla and Esselstyn 2015; Rannala and Yang 2017) and data sets of ∼10,000 loci (17 sequences per locus, 200 or 1,000 bp) from five species of gibbons (Carbone et al. 2014; Shi and Yang 2018). However, the algorithm was noted to have mixing problems in analysis of such large data sets.

### Mixing Problems and MCMC Diagnosis

Mixing problems in MCMC affect the efficiency, rather than the correctness, of the algorithm. A correct MCMC algorithm should visit the different models in proportion to their posterior probabilities. An efficient algorithm should jump between models frequently while an inefficient (lazy) algorithm may stay in one model for a long time before it jumps, and then remains in the new model for a long time before it jumps again. Both algorithms are correct in the sense that in the long run they both visit the models in proportion to their posterior probabilities. However, the lazy algorithm may be very inefficient as it takes an extremely long chain to generate reliable results because of the infrequent model jumps. The main symptom for poor mixing of the transmodel algorithm is that the chain gets stuck in one model (or a subset of models), and multiple runs (each over a finite but large number of iterations) produce different results. Mixing problems tend to be worse and occur more frequently for larger data sets but can occur even for small data sets.

Note that many of the standard MCMC diagnosis tools may not be very useful for transmodel algorithms. From our experience, comparing the results of multiple runs using different starting species trees may be the most effective way of ensuring the reliability of the results in transmodel algorithms. There are no hard rules for deciding how large a difference between runs is too large as this depends on the computing resources and the absolute running time, but accuracy at the percentage point seems desirable. Note that the variance of the estimate of the posterior model probability based on an MCMC sample of size *N* is *P*(1−*P*)/(*NE*), where *P* is the true posterior model probability, *E* is the efficiency of the MCMC sample, and *NE* is the effective sample size (ESS) (see, e.g., Yang 2014, p. 214). Thus to reduce the SE of the estimate by one half, one needs a 4-fold increase in the MCMC sample size.

### Summary and Perspectives

The multispecies coalescent provides a natural framework for accommodating incomplete lineage sorting and phylogenetic uncertainties at individual loci. It makes an efficient use of the information in the genomic sequence data and provides a powerful methodology for resolving challenging species phylogenies characterized by extremely short internal branches and large ancestral population sizes (Shi and Yang 2018). While easy species trees can be recovered using any methods including concatenation (although even in such cases concatenation creates biased estimates of divergence times and population sizes, Ogilvie et al. 2016), likelihood-based MSC methods have an advantage for difficult species trees generated during radiative speciation events. MSC methods are consistent (they will converge to the true species tree when the number of loci increases) and have higher efficiency (they recover the correct species tree with greater probability) than concatenation or summary methods (Ogilvie et al. 2016; Xu and Yang 2016; Shi and Yang 2018).

At the time of writing, the two versions of BPP (3.4 and 4.0) have nearly identical functionalities. Both implement the four different analyses described in Yang (2015): A00, A01, A10, and A11. Both include options for handling diploid sequences by analytically integrating out the different phase resolutions (Gronau et al. 2011), and for calculating the marginal likelihood (Bayes Factors) through thermodynamic integration and Gaussian quadrature (Lartillot and Philippe 2006; Rannala and Yang 2017). Both include check-pointing, which may be used to restart an aborted run. BPP4.0 has a computational advantage over 3.4: depending on the data and model, the speed difference can be several-fold. Much of this improvement results from savings on repeated calculations in the computation of the gene tree density (Rannala and Yang 2003). Both versions avoid repeated calculations of conditional probabilities in the computation of the sequence likelihood, but BPP3.4 does not implement similar savings on the gene tree density. More importantly, the redesign and reimplementation of the algorithms in BPP4.0 makes it easier for parallelization. We expect future improvements will be mostly made in BPP4.0.

Currently efforts are made to improve the MCMC algorithms for better mixing efficiency and to parallelize the code to improve the computational efficiency. Work is also under way to extend the JC69 mutation/substitution model to GTR+G (Yang 1994a, 1994b) and to relax the molecular clock (Xu and Yang 2016), so that the program can be used to estimate the species tree for distantly related species. The user is advised to check the program web site for future updates.
